# Weakly supervised deep learning for cutaneous squamous and basal cell carcinoma in whole‐slide histopathology

**DOI:** 10.1002/2056-4538.70082

**Published:** 2026-03-17

**Authors:** Anne Petzold, Anja Wessely, Stefan Schliep, Hong Jiang, Manuel Tran, Elias AT Koch, Tingying Peng, Hans Starz, Carola Berking, Carsten Marr, Markus V Heppt

**Affiliations:** ^1^ Department of Dermatology Deutsches Zentrum Immuntherapie (DZI), CCC Erlangen‐EMN, Bavarian Cancer Research Center (BZKF), Uniklinikum Erlangen, Friedrich‐Alexander‐Universität Erlangen‐Nürnberg (FAU) Erlangen Germany; ^2^ Laboratory for Dermatopathology, Oral Pathology and Molecular Pathology Dermpath München Munich Germany; ^3^ Institute of AI for Health Helmholtz Zentrum München–German Research Center for Environmental Health Neuherberg Germany; ^4^ Department of Mathematics Technical University of Munich Munich Germany; ^5^ Department of Physiology and Pharmacology Karolinska Institute Stockholm Sweden; ^6^ Helmholtz AI Helmholtz Zentrum München–German Research Center for Environmental Health Neuherberg Germany; ^7^ Department of Medicine III Ludwig‐Maximilian‐University Hospital Munich Germany; ^8^ DKTK German Cancer Consortium Munich Germany

**Keywords:** basal cell carcinoma, squamous cell carcinoma, skin neoplasms, deep learning, artificial intelligence, clinical pathology, computer‐assisted image interpretation

## Abstract

Distinguishing infiltrative basal cell carcinoma (BCC) from poorly differentiated cutaneous squamous cell carcinoma (cSCC) remains a significant histopathological challenge. Automated deep learning approaches hold promise for improving diagnostic reliability, yet robust external validation is essential. In this study, we developed a weakly supervised deep learning model to classify these diagnostically challenging subtypes and evaluated its generalizability across internal and external cohorts, as well as in comparison to a dermatopathology foundation model (HistoGPT). The model employed a multiple‐instance learning framework (CLAM) using the histopathology‐specific transformer Phikon for feature extraction from whole‐slide images. Slide‐level ground‐truth diagnoses from the collected images (*n* = 335, University Hospital Erlangen) were derived from routine clinical practice and re‐evaluated by two board‐certified dermatopathologists. Performance was assessed on an internal test set of 84 whole‐slide images (27 cSCC and 57 BCC) and two external datasets: Queensland cohort (*n* = 10, curated in‐distribution cases) and the COBRA cohort (*n* = 200, broad, partly out‐of‐distribution cases). Model discrimination was quantified using ROC curves, while accuracy, sensitivity, and specificity were reported alongside 95% Wilson confidence intervals (CIs). On the internal test set, the model achieved perfect classification [area under the receiver operating characteristic (AUC) = 1.0; 100% accuracy, sensitivity, and specificity]. Similarly, strong performance was observed in the Queensland cohort (AUC = 1.0), although limited by sample size. In the more heterogeneous COBRA cohort, discrimination remained high (AUC = 0.923, 95% CI 0.885–0.961), requiring threshold adjustment to correct for marked calibration shift (balanced accuracy 86.5% at Youden's *J*). Attention heatmaps highlighted histologically meaningful regions. In zero‐shot evaluation on the internal test set, HistoGPT achieved an overall accuracy of 77%, with high class‐wise sensitivity for BCC (98%, 95% CI 91–100) but markedly reduced sensitivity for cSCC (33%, 95% CI 19–52). Fine‐tuning a task‐specific classifier on the HistoGPT backbone substantially improved performance, achieving near‐perfect discrimination and 98% balanced accuracy. These findings demonstrate that weakly supervised deep learning enables highly accurate classification of diagnostically challenging BCC and cutaneous squamous cell carcinoma subtypes. However, reliable deployment across institutions necessitates careful calibration and domain adaptation, and even powerful foundation models such as HistoGPT benefit from targeted fine‐tuning to ensure robust performance in dermatopathology.

## Introduction

Basal cell carcinoma (BCC) and cutaneous squamous cell carcinoma (cSCC) are the two most common non‐melanoma skin cancers, together accounting for the vast majority of keratinocyte carcinomas worldwide [1]. While the overall prognosis is favorable, these tumors represent a significant clinical and socioeconomic burden due to their high incidence, frequent occurrence in cosmetically sensitive areas, and the need for histopathological confirmation prior to treatment [1, 2, 3].

Histopathology remains the gold standard for diagnosis. However, the distinction between infiltrative BCC and poorly differentiated cSCC can be challenging even for experienced dermatopathologists. These subtypes often display overlapping morphological features, and their reliable classification is crucial given differences in biological behavior, prognosis, and treatment strategies [4, 5, 6]. Automated approaches that support pathologists in differentiating these diagnostically demanding entities could therefore provide substantial clinical value.

Deep learning has shown considerable promise in histopathology, enabling slide‐level classification, detection of diagnostically relevant regions, and the integration of weakly supervised learning strategies that rely solely on global slide‐level labels, thereby bypassing the need for detailed region‐level annotations [7, 8]. Recent work has demonstrated that such models can reach or even exceed pathologist‐level performance in selected diagnostic tasks [8, 9]. Nevertheless, challenges remain, particularly regarding generalizability across institutions, domain shift due to staining or scanning variability, and the need for clinically interpretable outputs [10, 11].

To address these challenges, we developed a weakly supervised multiple instance learning (MIL) model to differentiate infiltrative BCC from poorly differentiated cSCC on whole‐slide images (WSIs). We validated this model on an internally held‐out test set and further assessed its performance on two independent external cohorts: a small curated subset from the University of Queensland [12] that matched our inclusion criteria, and a larger, more heterogeneous dataset from the COBRA repository [13], partly out of distribution. In addition, we benchmarked our data against HistoGPT [14], which, to our knowledge, currently represents the largest dermatopathology‐focused foundation model. We evaluated both zero‐shot performance and the improvements achieved through fine‐tuning a task‐specific classifier based on HistoGPT aggregator weights.

This work aims to provide a comprehensive evaluation of weakly supervised deep learning for diagnostically challenging skin cancer subtypes, highlight the impact of domain shift on model calibration, and explore the potential of foundation models as starting points for clinically applicable AI in dermatopathology.

## Materials and methods

### Ethics approval statement and consent to participate

Samples were used in accordance with ethical guidelines for the use of retrospective tissue samples at our institutions (ethics committee of the FAU Erlangen on October 5, 2023, approval number 22‐368‐Br).

### Dataset preparation

**Figure 1:**
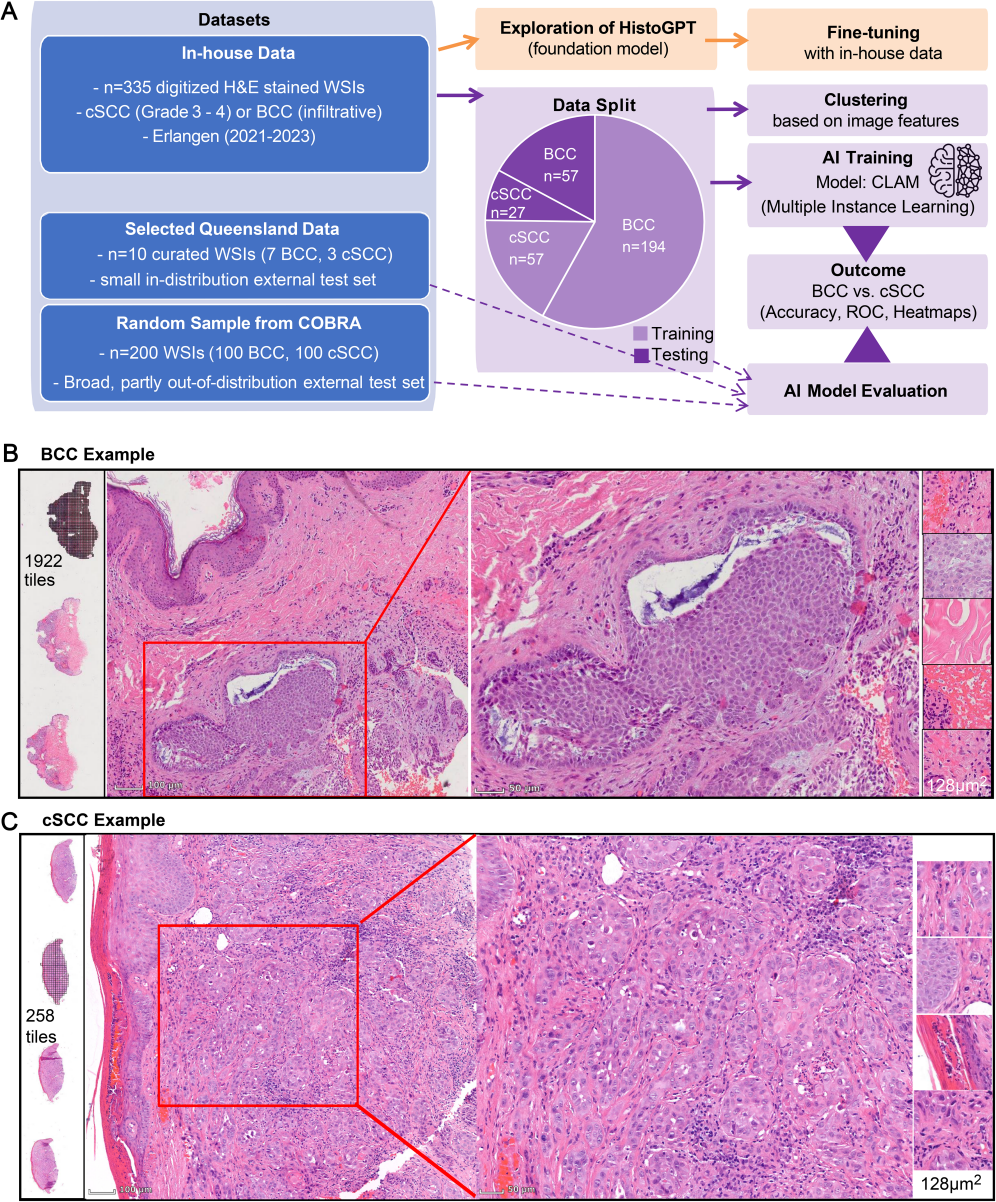
Overview of dataset preparation, model pipeline, and representative histology. (A) Flowchart summarizing the study design. Left: datasets included the in‐house cohort, the Queensland cohort, and the COBRA cohort. Middle: the in‐house cohort was split into training (75%) and held‐out test (25%) sets. Right: subsequent analyses comprised feature clustering as well as AI model training and evaluation. An additional pathway (orange panel) illustrates the exploration of the HistoGPT foundation model, including fine‐tuning a classifier based on HistoGPT‐L aggregator weights on the in‐house dataset. (B) Representative basal cell carcinoma (BCC) case from the in‐house cohort. The enlarged region highlights typical histological features: irregular islands of basaloid cells, peripheral palisading of tumor nuclei, clefting between tumor nests and adjacent stroma, and mitotic figures. Example tiles extracted at 256 × 256 pixels (0.5 μm/pixel) are shown. (C) Representative cutaneous squamous cell carcinoma (cSCC) case from the in‐house cohort. The enlarged region highlights a desmoplastic and cellular stroma surrounding infiltrative squamous cells, a characteristic histological feature of poorly differentiated cSCC. Example tiles are shown.

Digitized WSIs of poorly differentiated cSCC (grade 3–4) and infiltrative BCC were collected retrospectively from the dermatopathology unit of the University Hospital Erlangen, Germany, between 2021 and 2023 (in‐house data; Figure 1A). The cases were selected in a two‐tiered process. Diagnoses were derived from routine clinical practice as slide‐level labels, reviewed and confirmed by two board‐certified dermatopathologists, and served as the basis for case inclusion; unclear cases or cases with dissent among the pathologists in step 2 of the sample selection process were excluded. For cSCC, grade 3 (poorly differentiated, no to minimal keratinization) and grade 4 (no keratinization, undifferentiated) tumors were included; for clarity, these cases are collectively referred to as ‘poorly differentiated cSCC’ throughout this study. Infiltrative BCC was defined according to the presence of an infiltrative growth pattern in the original pathology report and included the infiltrative and sclerosing variants according to the current WHO classification. As commonly observed in routine practice, cases with mixed histological patterns were not excluded if an infiltrative component was present. No region‐of‐interest or patch‐level annotations were available. All slides were H&E‐stained and scanned with a Pannoramic 250 FLASH scanner.

Only one representative WSI per lesion was retained; duplicate slides or different histological levels from the same lesion were excluded to avoid redundancy and potential data leakage. When a WSI contained multiple tissue sections, a single representative section with minimal artifacts was manually selected using *SlideFlow Studio* [15]. No additional annotations were applied. To ensure unbiased evaluation, 25% of the in‐house slides were randomly held out as an internal test set.

Furthermore, two external cohorts were included to assess generalizability:

- Queensland cohort: Cases were selected from the publicly available Histopathology Non‐Melanoma Skin Cancer Segmentation Dataset [12] by two board‐certified dermatopathologists to match the internal inclusion criteria, including grade 3 poorly differentiated cSCC and infiltrative BCC, thereby providing a small in‐distribution test set.
- COBRA cohort: A balanced random sample of BCC and cSCC from the publicly available COBRA repository [13] served as a broader external test set. Detailed subtype annotations were not available for this dataset, precluding a more granular subtype‐based case selection. Cases were purposively sampled to obtain a balanced external test set that is comparable to the morphologic features of the cases included in the in‐house dataset. This cohort might still contain a broader mix of disease presentations than those used for training and was therefore considered partly out of distribution. From all datasets, image tiles of 256 × 256 pixels were extracted at 0.5 μm/pixel (128 μm field of view) (Figure 1B, C). Tiles with >60% background (grayscale fraction 0.6) were discarded. As an additional quality‐control step, the Blur Burden metric was applied to quantify out‐of‐focus or artifact‐rich tiles. Slides exceeding a Blur Burden of 5% triggered a warning in *SlideFlow* and were manually reviewed before inclusion. This quality–control–based approach was chosen instead of explicit stain normalization to preserve real‐world staining variability.

### Network architecture, training, and evaluation

We employed clustering‐constrained attention multiple‐instance learning (CLAM) [16], a weakly supervised deep learning approach that identifies diagnostically relevant regions *via* attention mechanisms and enables interpretable slide‐level heatmaps without requiring pixel‐level annotations. CLAM is publicly available as a Python package (https://github.com/mahmoodlab/CLAM).

Image tiles were first converted into features using Phikon [17], a vision transformer pretrained on ~40 million histopathology images, which produces a 768‐dimensional vector per tile. For each slide, the resulting feature vectors were grouped into bags that served as input for CLAM training. In addition, slide‐level feature representations were obtained by averaging tile vectors. These aggregated vectors were projected into two dimensions using uniform manifold approximation and projection (UMAP) [18] and subsequently clustered with DBSCAN [19] to explore latent structure within the in‐house dataset. Analyses were implemented in Python using *umap‐learn* and *scikit‐learn*.

Training was performed with FastAI [20] using a one‐cycle learning rate schedule [21] (initial learning rate 1 × 10^−4^, batch size 32, 32 epochs, threefold cross‐validation). Cross‐entropy loss and validation AUC were monitored during training. The final model was evaluated on the internal test set as well as on two independent external cohorts (Queensland and COBRA), outputting class probabilities and slide‐level attention heatmaps.

Model discrimination was quantified by the AUC with 95% confidence intervals (CIs; DeLong method [22]). For classification performance, we reported accuracy, sensitivity, and specificity with 95% Wilson score CIs [23], calculated both at the fixed threshold of 0.5 and at the Youden *J*‐optimal threshold [24]. For binary performance metrics, cSCC was defined as the positive class and BCC as the negative class. To assess calibration under domain shift, performance at the 0.5 threshold was compared with the Youden threshold.

All training and inference were carried out with Slideflow [15] on a NVIDIA GeForce RTX 4070 GPU. Statistical analyses were performed in R using the *pROC*, *PRROC*, and *binom* packages.

### Exploration and fine‐tuning of HistoGPT

HistoGPT is a recently introduced large‐scale foundation model for digital pathology, trained on multi‐center histopathology data and designed to combine strong classification performance with integrated reporting capabilities [14]. To benchmark our in‐house dataset against this model, we employed the use of HistoGPT, specifically the large variant HistoGPT‐L. First, we conducted zero‐shot inference using the pre‐trained model with the prompt ‘Final diagnosis’, in order to establish a baseline performance for BCC and cSCC classification without task‐specific training. Subsequently, we fine‐tuned a HistoGPT‐L‐based classifier on our in‐house data to adapt the model to the target task. Specifications on the fine‐tuning are provided in supplementary material, File S1.

## Results

### Dataset characteristics

We collected 335 digitized H&E‐stained WSIs, each derived from a different lesion: 84 cSCC samples from 75 patients and 251 BCC samples from 240 patients. Among cSCC tumors, 82.1% occurred in men, while 60.2% of BCC tumors were found in men. The mean age of cSCC patients was 79.8 years (range 51–96), and for BCC patients 72.4 years (range 34–99). The majority of both tumor types were located in the head and neck region (cSCC: 86.9%; BCC: 80.5%). Most cSCC lesions were poorly differentiated, with 84.5% classified as G3. The baseline characteristics of the in‐house cohort are summarized in Table 1.

**Table 1:** Baseline characteristics of the in‐house dataset

	cSCC (*N* = 84 samples of 75 patients)	BCC (*N* = 251 samples of 240 patients)
Sex, *N* (%)	Male: 69 (82.1%) Female: 15 (17.9%)	Male: 151 (60.2%) Female: 100 (39.8%)
Age in years, mean (range)	79.8 (51–96)	72.4 (34–99)
Localization	Head and neck: 73/84 = 86.9% Trunk: 6/84 = 7.1% Extremities: 5/84 = 6.0%	Head and neck: 202/251 = 80.5% Trunk: 32/251 = 12.7% Extremities: 16/251 = 6.4% n.a.: 1/251 = 0.4%
Grade of differentiation in cSCC (G3 or G4)	G3: 71/84 = 84.5% G4: 13/84 = 15.5%	

BCC, basal cell carcinoma; cSCC, cutaneous squamous cell carcinoma; G3, grade 3; G4, grade 4; n.a., not available.

For evaluation, we randomly set aside an internal test set of 84 WSIs (27 cSCC and 57 BCC; 25.1%), while 251 WSIs (57 cSCC and 194 BCC; 74.9%) were used for training (Figure 1A).

For further evaluation, we included two external cohorts. The Queensland cohort comprised 10 WSIs (7 BCC and 3 cSCC) from the University of Queensland dataset [12]. In addition, 200 WSIs (100 BCC and 100 cSCC) were sampled from the COBRA repository [13].

### Clustering, training, and internal evaluation

**Figure 2:**
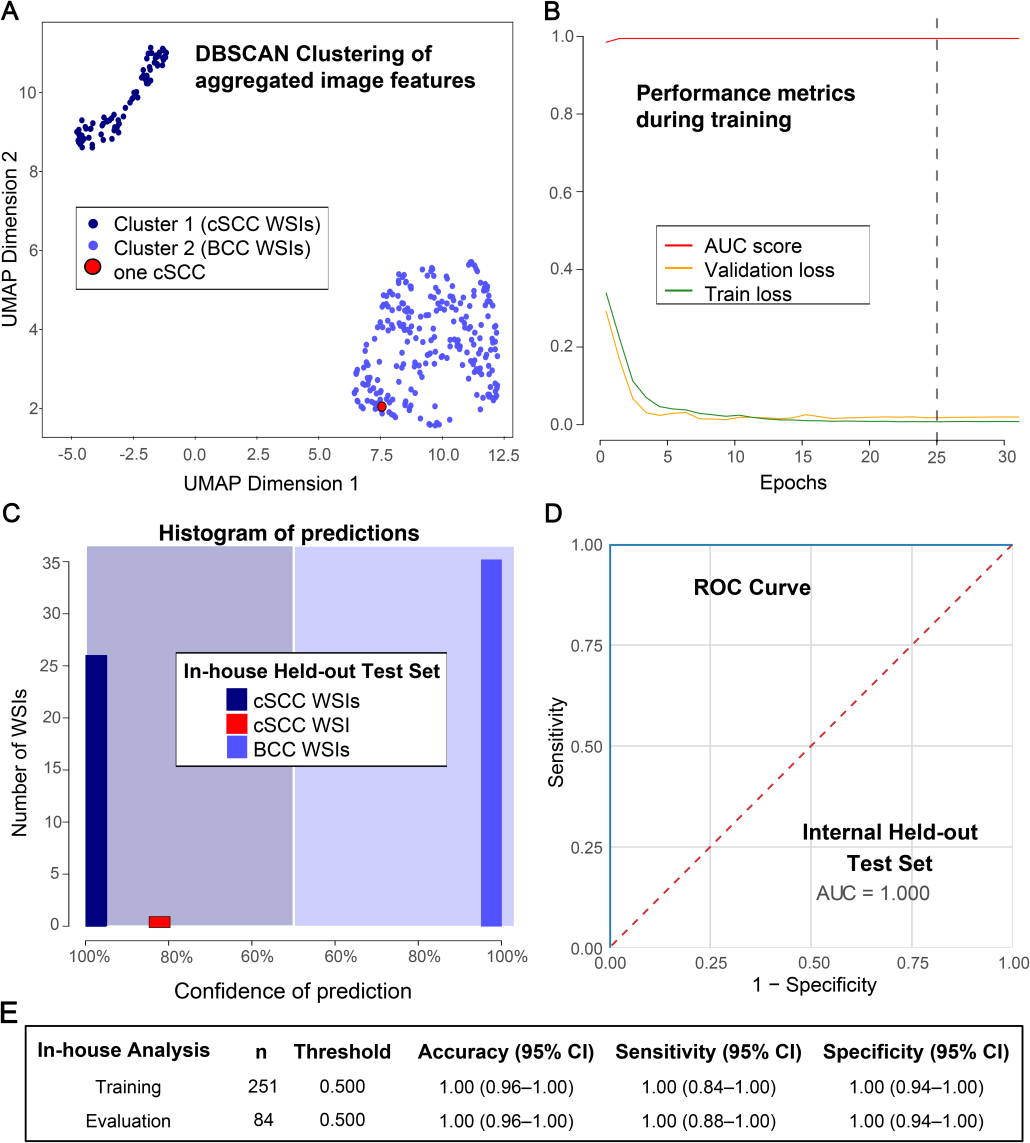
Internal performance of the CLAM model. (A) Unsupervised clustering of aggregated slide‐level feature vectors using UMAP and DBSCAN revealed a clear separation of cutaneous squamous cell carcinoma (cSCC, dark blue) and basal cell carcinoma (BCC, light blue) WSIs. One cSCC (red) clustered within the BCC group. (B) Training metrics in k‐fold 1 over 32 epochs. The model selected for evaluation was taken from epoch 25, corresponding to the lowest validation loss. AUC, area under the receiver operating characteristic (ROC) curve. (C) Histogram of predictions on the internal test set. All BCC WSIs and all but one cSCC WSI were classified with high confidence (>0.95). The single cSCC having a prediction of 84.6% corresponded to the outlier identified in the clustering analysis (panel A). (D) ROC curve for the internal test set, demonstrating perfect discrimination [AUC = 1.0 (95% CI 1.000–1.000)]. (E) Summary table reporting accuracy, sensitivity, and specificity relative to cSCC for both training and internal evaluation.

Clustering of the aggregated slide‐level feature vectors revealed two distinct groups (Figure 2A). Cluster 1, located in the upper left corner, consisted exclusively of cSCC samples, while Cluster 2 contained all BCC samples. One cSCC slide, however, clustered with the BCC group.

During model training (k‐fold 1), the best‐performing epoch was 25, corresponding to the lowest validation loss (1.36 × 10^−11^; Figure 2B). At this point, the training loss was 0.00044, the validation AUC reached 1.0, and classification accuracy was 100% (95% CI 96–100). Comparable outcomes were observed in folds 2 and 3, each achieving an AUC of 1.0 and 100% accuracy; the model from fold 1 slightly outperformed the others and was therefore selected for downstream evaluation.

**Figure 3:**
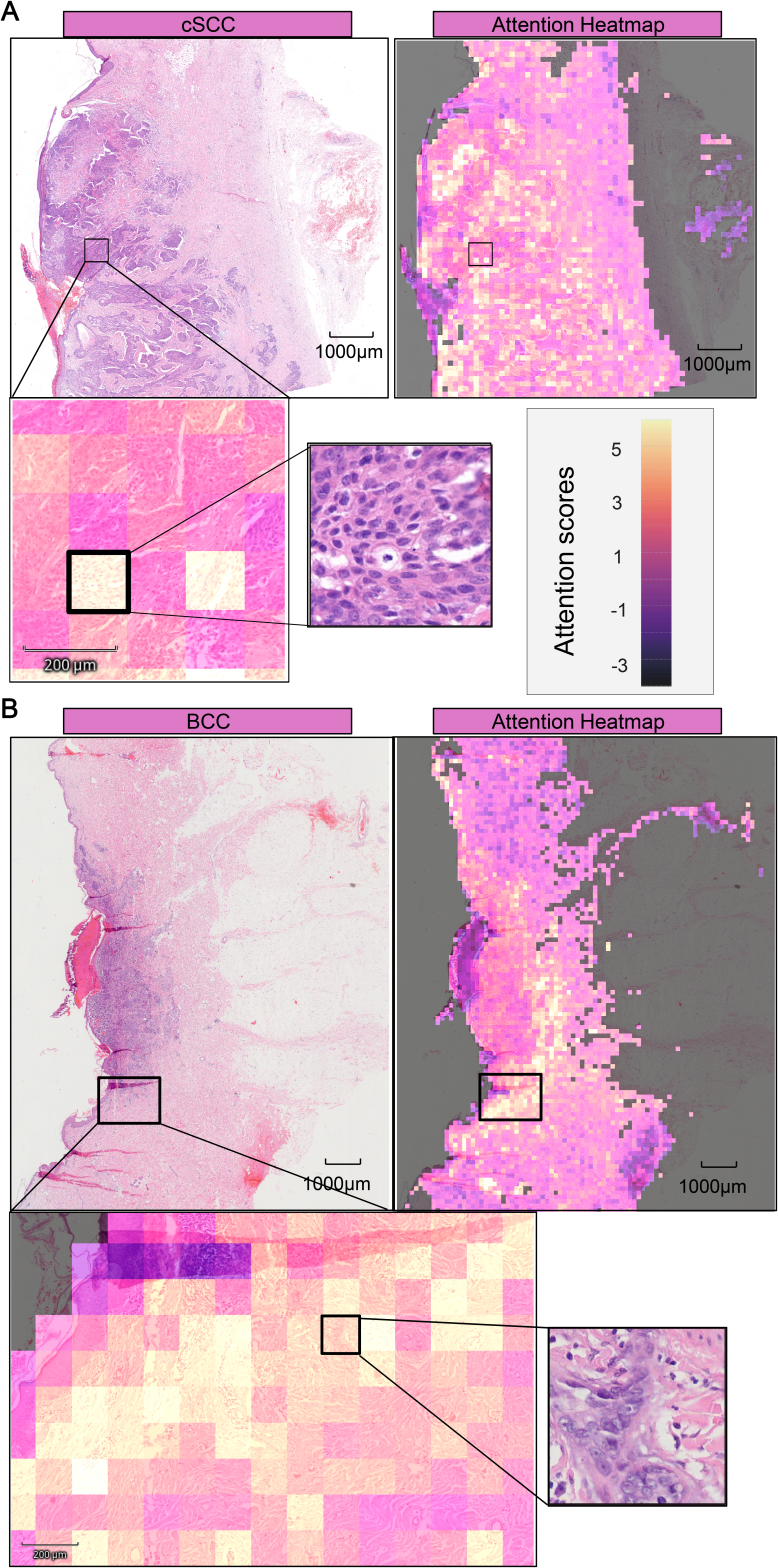
Representative attention heatmaps of cSCC and BCC. (A) Example of a correctly predicted cSCC slide from the internal test set. The attention heatmap indicates tumor regions with high attention. The highlighted tile in magnification depicts infiltrative malignant squamous cells and desmoplastic stroma, typical features of cSCC. (B) Example of a correctly predicted BCC slide from the internal test set. High attention was concentrated within infiltrative tumor cells. The highlighted tile in magnification shows a strand of basaloid cells with mitotic figures, typical of infiltrative BCC.

When applied to the held‐out internal test set (*n* = 84), prediction scores were highly confident, with probabilities above 0.95 for all but one WSI (Figure 2C). This exception corresponded to the same cSCC slide identified as an outlier in the clustering analysis (Figure 2A), which yielded a prediction score of 0.846 but was nevertheless correctly classified. The model again achieved perfect discrimination (AUC = 1.0, 95% CI 1.0–1.0; Figure 2D). Accuracy, sensitivity, and specificity were each 100% (95% CI 95.6–100, 87.5–100, and 93.7–100, respectively; Figure 2E). Representative attention heatmaps for correctly classified cSCC and BCC samples are provided in Figure 3.

Feature extraction was performed once prior to training and required approximately 2–3 h for the full dataset. Subsequent model training required about 1.5 h. Inference for a single WSI was performed on the order of seconds to under 1 min, depending on slide size.

### Attention heatmaps

To illustrate model interpretability, representative attention heatmaps are shown in Figure 3. In a cSCC case (Figure 3A), tiles with the highest attention scores were localized within tumor regions. The highlighted example tile depicted infiltrative atypical squamoid cells and surrounding desmoplastic stroma, a known feature of poorly differentiated cSCC [6] In a BCC case (Figure 3B), attention concentrated within infiltrative tumor strands, and the highlighted example tile showed a strand of basaloid cells with visible mitotic figures, which are characteristic of infiltrative BCC [4]. These examples suggest that, at least in selected tiles, the model's predictions were driven by histologically meaningful regions.

### External evaluation with Queensland and COBRA data

**Figure 4:**
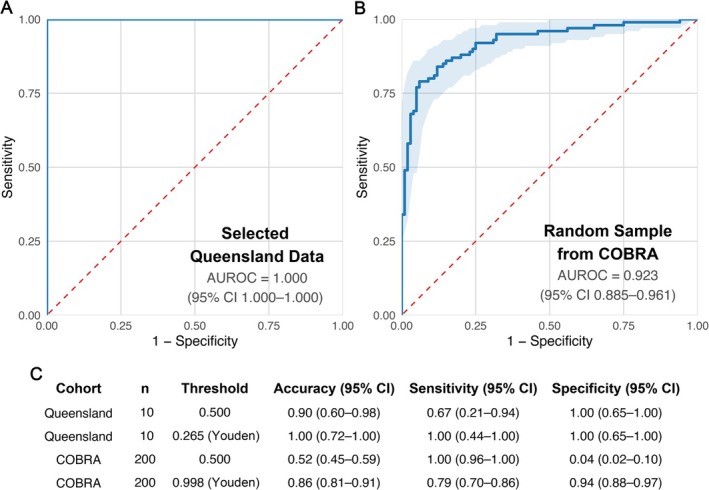
External evaluation of the CLAM model. (A) ROC curve for the curated Queensland cohort (*n* = 10), showing perfect discrimination (AUC = 1.0). (B) ROC curve for the COBRA cohort (*n* = 200), demonstrating strong discriminatory ability (AUC = 0.923). (C) Performance metrics including accuracy, sensitivity, and specificity relative to cSCC with 95% confidence intervals for both external cohorts, reported at a fixed threshold of 0.5 and at the Youden J‐optimal threshold.

In the Queensland subset (*n* = 10), the model achieved perfect discrimination (AUC = 1.0, 95% CI 1.0–1.0; Figure 4A). At the fixed 0.5 threshold, all seven BCCs were correctly classified (100%, 95% CI 64.6–100), while two of three cSCCs were correctly identified (66.7%, 95% CI 20.8–93.9), yielding an overall accuracy of 90% (95% CI 59.6–98.2). At the Youden threshold (0.27), all cases were correctly classified (Figure 4C). Given the small sample size, CIs were wide, and the perfect AUC should be interpreted with caution.

In the COBRA cohort (*n* = 200), the model achieved an AUC of 0.923 (95% CI 0.885–0.961; Figure 4B). At the fixed 0.5 threshold, accuracy was 52.0% (95% CI 45.1–58.8), driven by very high sensitivity (100%, 95% CI 96.3–100) but very low specificity (4.0%, 95% CI 1.6–9.8). After threshold adjustment with Youden's *J* (0.998), accuracy improved to 86.5% (95% CI 81.1–90.6), with balanced sensitivity (79.0%, 95% CI 70.0–85.8) and specificity (94.0%, 95% CI 87.5–97.2; Figure 4C). This reflects strong discriminative power but a marked calibration shift in this external dataset.

A pooled Youden threshold across all external and internal test cohorts was not computed, as the cohorts differed substantially in size and acquisition characteristics. Such pooling would be dominated by the larger COBRA dataset and potentially obscure cohort‐specific calibration effects.

### Exploration of HistoGPT and fine‐tuning a classifier based on HistoGPT‐L

**Figure 5:**
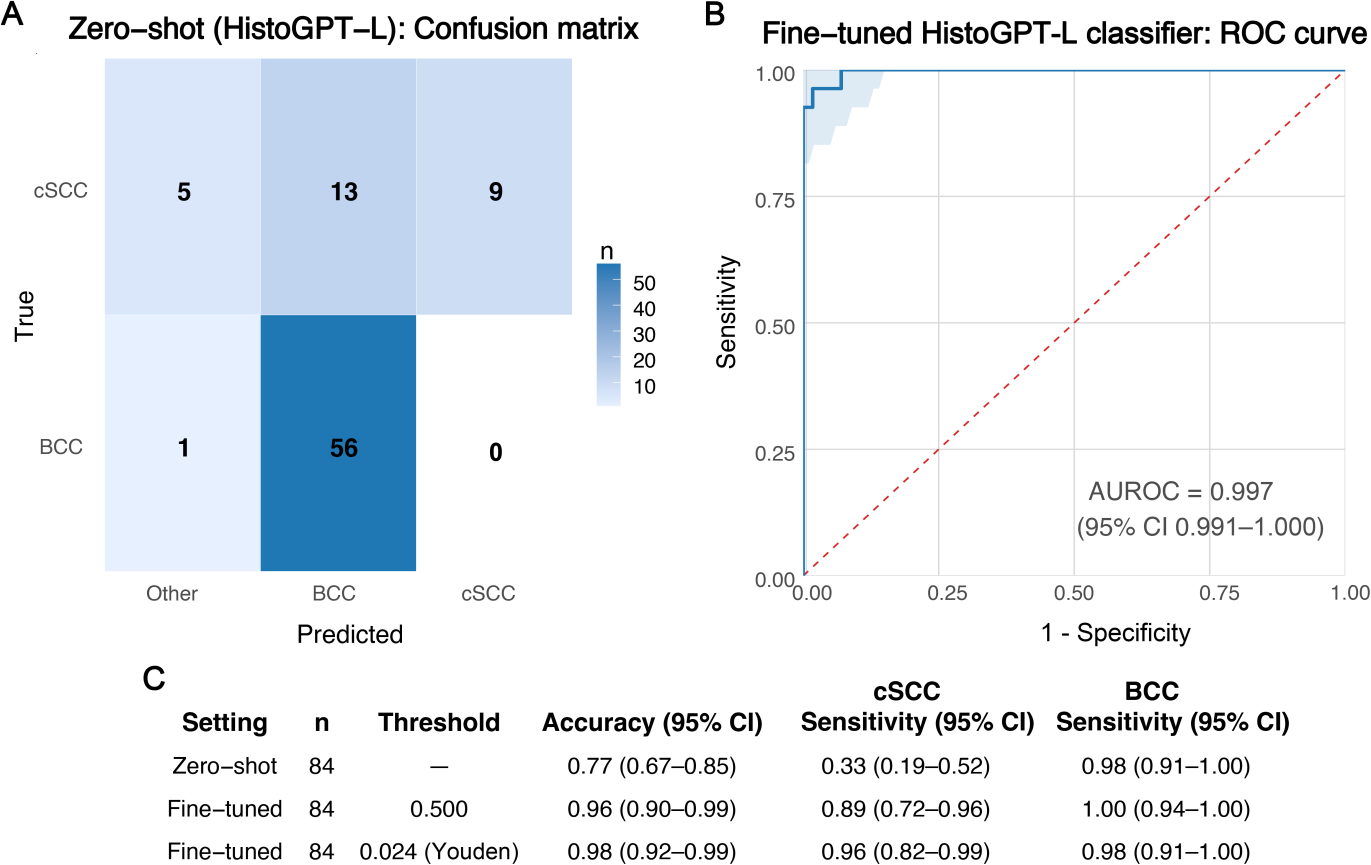
Comparison of zero‐shot HistoGPT‐L performance and the fine‐tuned HistoGPT‐L–based classifier. (A) Confusion matrix of the zero‐shot evaluation using HistoGPT‐L with the prompt ‘Final diagnosis’. Predictions included BCC, cSCC, or unrelated diagnoses (Other), the latter of which were always counted as incorrect. While BCC cases were largely recognized, cSCC was frequently misclassified or assigned to Other. (B) Receiver operating characteristic (ROC) curve of the fine‐tuned classifier based on HistoGPT‐L. Fine‐tuning markedly improved classification performance. (C) Summary table of performance metrics (accuracy, cSCC, and BCC sensitivity with 95% Wilson confidence intervals). Results are shown for the zero‐shot experiment and for the fine‐tuned model at both the fixed 0.5 threshold and the Youden J‐optimal threshold.

For the zero‐shot evaluation, HistoGPT‐L predictions were categorized as BCC, cSCC, or ‘Other’ (unrelated diagnoses). Of 27 cSCC cases, 9 were correctly classified, 13 were misclassified as BCC, and 5 received unrelated diagnoses (sensitivity 33%, 9/27; 95% CI 19–52). Of 57 BCC cases, 56 were correctly classified, with 1 assigned an unrelated diagnosis (class‐wise sensitivity 98%, 56/57; 95% CI 91–100). No BCC case was misclassified as cSCC, corresponding to a specificity of 100% for cSCC detection. Predictions categorized as ‘Other’ were counted as incorrect. The model's overall accuracy was 77% (65/84; 95% CI 67–85) with a consistent tendency to misclassify poorly differentiated cSCC cases (Figure 5A).

Fine‐tuning the HistoGPT‐L‐based classifier on our in‐house dataset substantially improved performance. The fine‐tuned classifier reached an AUC of 0.997 (95% CI 0.991–1.000) (Figure 5B). At the fixed threshold of 0.5, accuracy was 96% (95% CI 90–99), with a sensitivity of 89% (95% CI 72–96) and a specificity of 100% (95% CI 94–100). Applying the Youden *J*‐optimal threshold further improved balance, yielding an accuracy of 98% (95% CI 92–99), sensitivity of 96% (95% CI 82–99), and specificity of 98% (95% CI 91–100) (Figure 5C).

## Discussion

In this study, we trained and evaluated a weakly supervised MIL model (CLAM) to distinguish between infiltrative BCC and poorly differentiated cSCC, two histological subtypes that are often difficult to differentiate. This deliberate focus underscores the clinical relevance of our work: while nodular BCC and highly differentiated cSCC are more easily distinguishable, the subtypes we targeted remain a diagnostic challenge.

The internal evaluation demonstrated excellent performance, with perfect separation of BCC and cSCC cases. Notably, even an unsupervised clustering of aggregated slide‐level features already produced a clear dichotomy between the two tumor types. This simplicity in separation suggests that the selected cases carried distinct morphological signals. Only one cSCC slide clustered with the BCC group; this case was re‐reviewed by two dermatopathologists and confirmed as cSCC. This discrepancy likely reflects the limitations of unsupervised clustering based on globally averaged features and dimensionality reduction. In contrast, the weakly supervised MIL model leverages contextual and higher‐order slide‐level information during training, enabling correct classification of this case despite its ambiguous feature representation.

External evaluation provided important insights into the model's generalizability. In the small curated Queensland dataset, which was matched to our inclusion criteria, performance was excellent with an AUC of 1.0, although the limited sample size restricts the robustness of this finding. In contrast, the COBRA dataset is broader and may include subtypes not represented in training. Here, the model still achieved strong discriminatory power (AUC = 0.923), but raw predictions at the naive threshold showed high sensitivity and very low specificity – indicating a calibration shift. Only after threshold adjustment were balanced metrics restored, illustrating the impact of domain differences. These differences likely stem from multiple sources: (1) inclusion of diagnostic subtypes in COBRA that lie outside our training domain, and (2) differences in image resolution and file format – our in‐house WSIs were high‐resolution .svs files (~2 GB), while external datasets were compressed .tiff images (~60 MB). Variations in scanning resolution and compression introduce artifacts and domain shift that degrade performance, even when underlying histological features remain equivalent [10].

Attention heatmaps enhanced interpretability by illustrating which tissue regions the model focuses on. In the presented examples, high‐attention tiles corresponded to histologically meaningful features, such as infiltrative squamoid cells with desmoplastic stroma in cSCC or basaloid tumor strands with mitotic figures in BCC, suggesting class‐specific attention patterns. Notably, high‐attention regions frequently extended into the peritumoral stroma, consistent with the importance of architectural growth patterns and tumor–stroma interactions in the histopathological distinction of these entities. However, not all high‐attention regions necessarily depict pathognomonic structures, and interpretability should therefore be regarded as illustrative rather than absolute.

From a practical standpoint, computational efficiency is an important consideration for clinical translation. In this study, model training and feature extraction were performed offline and are not intended to be part of routine diagnostic workflows. While inference was accelerated using GPU hardware, inference on standard CPU‐based systems commonly available in clinical environments remains feasible, with typical runtimes on the order of 1–2 min per WSI. As such, inference time is unlikely to constitute a major bottleneck for clinical use, particularly when weighed against slide scanning, data transfer, and routine diagnostic turnaround times.

The collaboration with the Helmholtz Institute of AI for Health to evaluate HistoGPT‐L, a pathology foundation model, further underscores the value of domain‐specific adaptation. In the zero‐shot setting, HistoGPT‐L achieved high class‐wise sensitivity for BCC but consistently underperformed for poorly differentiated cSCC, resulting in an overall accuracy below that of our weakly supervised CLAM model. This suggests that, while such models contain rich generalizable representations, they may not reliably capture the subtleties of diagnostically challenging subtypes out of the box. Importantly, fine‐tuning in this study was performed using a newly trained binary classification head built on the frozen HistoGPT‐L backbone, while the original model with its broad multi‐disease diagnostic capabilities and report generation remained unchanged. The substantial performance gains observed after fine‐tuning therefore reflect targeted, task‐specific adaptation rather than an improvement or degradation of HistoGPT‐L's original functionality. Through this adaptation, the imbalance observed in the zero‐shot setting was largely eliminated, resulting in balanced, near‐perfect performance. These results emphasize that domain‐specific fine‐tuning is indispensable when applying foundation models to nuanced diagnostic scenarios.

To our knowledge, this study represents the first deep learning–based investigation to explicitly address the binary histopathological distinction between BCC and cSCC on WSIs. While this differential diagnosis is clinically highly relevant – particularly for infiltrative BCC and poorly differentiated or undifferentiated cSCC – it has not been directly evaluated in prior computational pathology studies.

Existing work in non‐melanoma skin cancer has predominantly focused on related but distinct binary classification tasks. Geijs *et al* [25], Xu *et al* [26], Kimeswenger *et al* [27], and O'Brien *et al* [28] have investigated BCC versus non‐BCC classification and reported high performance for BCC detection across diverse datasets. These approaches generally frame the task as tumor detection against heterogeneous benign tissue or histological mimics, rather than as a direct differential diagnosis between two malignant epithelial entities.

Similarly, Rios‐Duarte *et al* [29] have addressed SCC versus non‐SCC classification primarily in the context of tumor detection and domain transfer, rather than fine‐grained subtype discrimination. As a result, the diagnostic task in these studies is substantially less constrained than the direct distinction between BCC and cSCC.

In addition to differences in task definition, prior studies often rely on extensive patch‐level annotations, fully supervised training paradigms, and single‐cohort evaluations. In contrast, our approach employs a weakly supervised MIL framework trained solely on slide‐level routine diagnoses and systematically evaluates generalizability across two independent external cohorts. This positions the present work as a focused examination of a high‐difficulty, clinically meaningful differential diagnosis, extending prior non‐melanoma skin cancer studies toward scenarios where automated decision support may be most valuable.

Beyond individual studies, recent meta‐analytical resources such as the HistoPathExplorer platform [30] provide a comprehensive overview of AI applications in histopathology. Such initiatives not only contextualize our findings within the broader field but also highlight methodological heterogeneity and translation barriers that need to be addressed to bring computational pathology into routine practice.

Several limitations warrant consideration. Our in‐house dataset was monocentric (single institution, scanner, and subtype range), which limits external validity. The external cohorts, while helpful, are limited in scale and subtype representation. Differences in scanning hardware, image file format, and compression may induce domain shifts significant enough to degrade performance [10], consistent with upstream findings on the impact of scanner‐induced shifts.

In addition, this study did not include quantitative annotations of subtype proportions or tumor area composition within individual WSIs. Poorly differentiated cSCC and infiltrative BCC frequently coexist with additional histological components, such as well‐differentiated cSCC areas, overlying or adjacent actinic keratosis, or nodular/superficial BCC components. As a result, the contribution of such features to individual model predictions cannot be disentangled in a weakly supervised setting. However, this reflects the reality of routine diagnostic practice, where slides are rarely composed of homogeneous tumor patterns. Accordingly, the present results should be interpreted as slide‐level diagnostic discrimination under real‐world conditions rather than as classification of isolated or overly dissected tumor subregions. Similarly, attention heatmaps were employed exclusively for qualitative illustration. No quantitative or systematic analysis of attention patterns was performed, and interpretability should therefore be regarded as supportive rather than definitive.

## Conclusion

Weakly supervised learning enabled robust differentiation of challenging subtypes of keratinocyte carcinomas, supported by interpretable visualizations and validated on both in‐distribution and partly out‐of‐distribution external cohorts. However, domain‐specific calibration, image standardization, and larger‐scale validation remain essential for reliable clinical translation. Foundation models offer a powerful starting point, but require careful fine‐tuning to achieve robust performance across tumor subtypes and institutions.

## Author contributions statement

AP: data curation, formal analysis, investigation, methodology, software, validation, visualization, writing – original draft. AW: writing – review and editing. SS: writing – review and editing. HJ: investigation, software, validation, writing – review and editing. MT: investigation, software, validation, writing – review and editing. EATK: conceptualization, data curation, investigation, writing – review and editing. TP: supervision, writing – review and editing. HS: supervision, writing – review and editing. CB: project administration, supervision, resources, validation, writing – review and editing. CM: supervision, writing – review and editing. MVH: conceptualization, data curation, project administration, supervision, resources, validation, writing – review and editing.

## Supporting information


**File S1.** Training specifications for fine‐tuning the HistoGPT‐L based classifier

## Data Availability

All study‐related data and programming code are stored at the Uniklinikum Erlangen, Friedrich‐Alexander‐Universität Erlangen‐Nürnberg (FAU), 91054 Erlangen, Germany, and are available upon reasonable request.
