# Supplementary material for: Weakly supervised deep learning for cutaneous squamous and basal cell carcinoma in whole‐slide histopathology
Source: J Pathol Clin Res. 2026 Mar 17;12(2):e70082. doi: 10.1002/2056-4538.70082 (PMC13093612; doi:10.1002/2056-4538.70082)
Supplement: Supplementary file 1 — File S1. Training specifications for fine‐tuning the HistoGPT‐L based classifier [file CJP2-12-e70082-s001.pdf]

Weakly supervised deep learning for cutaneous squamous and basal cell carcinoma in whole-slide histopathology

A Petzold *et al*. *J Pathol Clin Res* https://doi.org/10.1002/2056-4538.70082

File S1. Training specifications for fine-tuning the HistoGPT-L based classifier

Model architecture

Component	Specification
Backbone	Pretrained HistoGPT-L aggregator
Input features	Patch embeddings (1024-dim) + 3D spatial coordinates
Positional embedding	NaViTEmbedding (d_model = 1024, max_len = 1024, patch_size = 512)
MIL classifier	CancerClassifier with gated attention pooling
Attention framework	FlashPerceiver, 6 layers, 16 heads, 1536-dim latent, 640 latent tokens
Normalization	FusedRMSNorm (eps = 1e-5)
Classification head	Linear layer (1536 → 2 classes)

Data preparation

- Features extracted and stored in HDF5 format (features: $N \times 1024$; coordinates: $N \times 3$).
- Maximum 1,000 patches per slide used during training and inference.
- Batch size = 1 slide per iteration.

Training setup

Parameter	Specification
Cross-validation	Stratified 5-fold
Optimizer	AdamW (betas = 0.9, 0.95; weight decay = 0.05)
Learning rate schedule	Warmup + cosine decay (start 0.0 → peak 5e-5 → final 1e-6)
Precision	bf16 mixed precision
Gradient accumulation	16 batches, clipping = 1.0
Early stopping	Patience = 10 epochs, delta = 0.001
Selection criterion	Best validation AUROC checkpoint retained

Evaluation metrics: AUROC**,** accuracy, sensitivity, specificity

Reproducibility

- Pretrained backbone weights initialized from HistoGPT-L
- Fine-tuned model checkpoint: https://drive.google.com/file/d/1DRg7pk1PUL66EziffK3AJW51iLGjJUOD/view?usp=drive_link.
